# Supplementary material for: Splice-Junction-Based Mapping of Alternative Isoforms in the Human Proteome
Source: Cell Rep. Author manuscript; Available in PMC 2020 Jan 15. (PMC6961840; doi:10.1016/j.celrep.2019.11.026)
Supplement: 2 [file NIHMS1546469-supplement-2.zip › DF1/PXD006675/PulmonaryArtery/PulmonaryArtery_13_PALLD_FKEDLLNNGQPIYWFK.pdf]

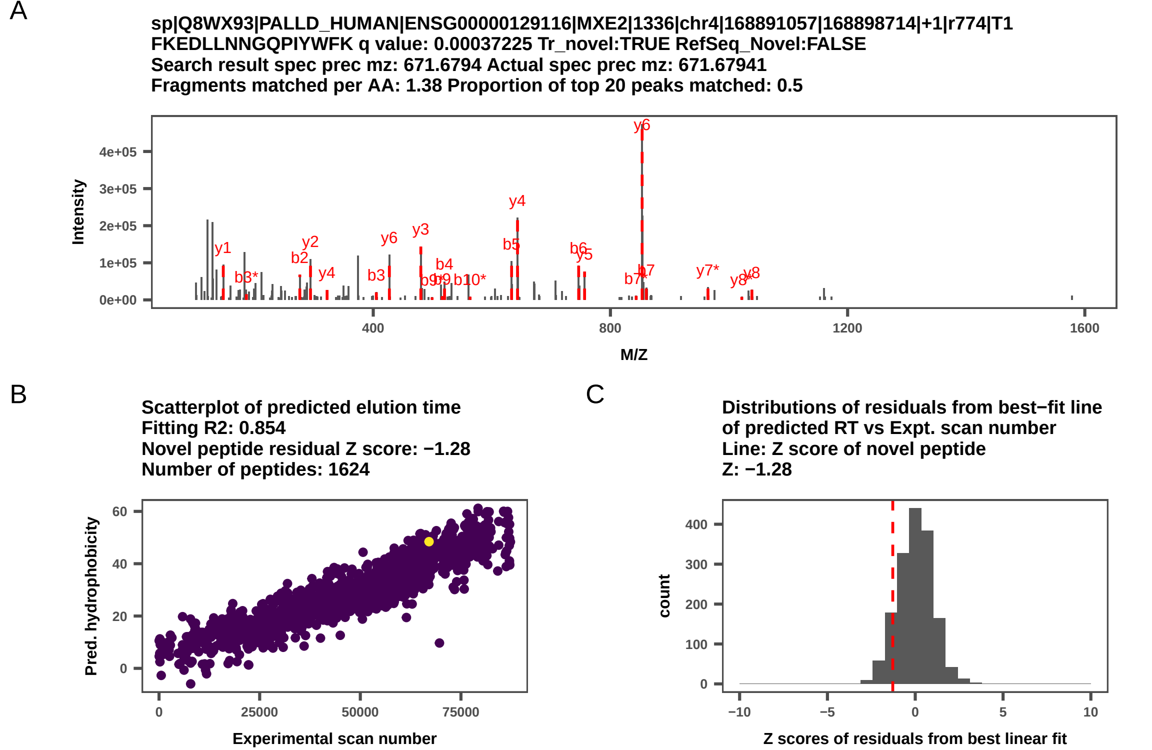
A
sp|Q8WX93|PALLD_HUMAN|ENSG00000129116|MXE2|1336|chr4|168891057|168898714|+1|r774|T1
FKEDLLNNGQPIYWFK q value: 0.00037225 Tr_novel:TRUE RefSeq_Novel:FALSE
Search result spec prec mz: 671.6794 Actual spec prec mz: 671.67941
Fragments matched per AA: 1.38 Proportion of top 20 peaks matched: 0.5
Intensity
4e+05
3e+05
2e+05
1e+05
0e+00
M/Z
400
800
1200
1600
y1
y2
b2
b3*
y4
y6
b3
y3
b4
b10*
y4
b5
b6
y5
y6
b7
y7*
y8
B
Scatterplot of predicted elution time
Fitting R2: 0.854
Novel peptide residual Z score: −1.28
Number of peptides: 1624
Pred. hydrophobicity
60
40
20
0
0
25000
50000
75000
Experimental scan number
C
Distributions of residuals from best−fit line
of predicted RT vs Expt. scan number
Line: Z score of novel peptide
Z: −1.28
count
400
300
200
100
0
−10
−5
0
5
10
Z scores of residuals from best linear fit
